# Strain-based method for fatigue failure prediction of additively manufactured lattice structures

**DOI:** 10.1038/s41598-023-49846-z

**Published:** 2023-12-20

**Authors:** Antonio Coluccia, Giorgio De Pasquale

**Affiliations:** Smart Structures and Systems Laboratory, Department of Mechanical and Aerospace Engineering, Politecnico di Torino, Corso Duca degli Abruzzi 24, 10129 Turin, Italy

**Keywords:** Mechanical engineering, Aerospace engineering

## Abstract

Lattice structures find application in numerous technological domains, including aerospace and automotive industries for structural components, biomedical sector implants, and heat exchangers. In many instances, especially those pertaining to structural applications, fatigue resistance stands as a critical and stringent requirement. The objective of this paper is to advance the analysis of fatigue failure in additively manufactured lattice structures by introducing a predictive fatigue failure model based on the finite element (FE) method and experimentally validating the results. The model utilizes linear homogenization to reduce computational effort in FE simulations. By employing a strain-based parameter, the most critical lattice cell is identified, enabling the prediction of fatigue crack nucleation locations. The Crossland multiaxial fatigue failure criterion is employed to assess the equivalent stress, furnishing the fatigue limit threshold essential for predicting component failure. Inconel 625 specimens are manufactured via the laser-based powder bed fusion of metals additive manufacturing process. In order to validate the model, cantilevers comprising octa-truss lattice cells in both uniform and graded configurations undergo experimental testing subjected to bending loads within the high cycle fatigue regime. The proposed methodology effectively forecasts the location of failure in seventeen out of eighteen samples, establishing itself as a valuable tool for lattice fatigue analysis. Failure consistently manifests in sections of uniform and graded lattice structures characterized by the maximum strain tensor norm. The estimated maximum force required to prevent fatigue failure in the samples is 20 N, based on the computed Crossland equivalent stress.

## Introduction

Lattice structures have garnered significant attention in various engineering sectors, including aerospace, biomedical, and general mechanics, over the past few decades. These structures, considered a subset of cellular solids, have gained recognition for their exceptional mechanical properties^1,2^, functionality^3^, and lightweight nature. Lattices exhibit typical characteristics of cellular solids, manifesting as reticulated, truss, or shell structures comprised of repeating unit cells. In the context of such structures, and composite materials sharing similar multi-phase compositions, an essential topological concept is the representative volume element (RVE). The RVE represents a defined volume capable of encapsulating a single (or multiple) repeating unit cells, providing a reasonably accurate depiction of macroscale material properties. The employment of lattice structures offers significant advantages in design activities driven by specific requirements^4,5^. The topology and distribution of cells can be effectively managed using optimization algorithms or process-driven controls. Optimization, as extensively explored in scientific literature^6,7^, plays a crucial role in enhancing lattice structures.

The design process is inherently constrained by the chosen fabrication method, particularly additive manufacturing (AM) processes. While AM enables the generation of highly intricate geometries, challenges related to part resolution may arise, particularly in serial production with statistical deviations impacting performance. These deviations can have adverse effects on the reliability of lattice structures, stemming from dimensional uncertainty, surface roughness, porosity, lack of fusion, stress intensification at corners, and impractical mechanical treatment of surfaces. Consequently, lattice structure characterization assumes critical importance^8^ across different mechanics disciplines, enabling the investigation of defects' impact on mechanical properties and methods for mitigating them.

Lattices are utilized not only for their structural properties but also as components for heat management in various scenarios. Honeycomb lattices enhancing the heat transportation of nanoparticles have been investigated showing good results^9^. A predictive model for heat flow around a C/SiC composite pyramidal lattice structure is proposed by Wang et al.^10^. Furthermore, effect of lattice presence in tubes on fluid flow and heat transfer enhancement, particularly with supercritical CO2, have been explored by Shi et al.^11^. Additively manufactured periodic structures have been explored within the domain of energy generation^12^, and three-dimensional bio-inspired nanostructures have been utilized to enhance the performance of catalysis engineering reactors^13^. Biomedical engineering is an important domain where lattice structures find extensive application, owing to their exceptional osteointegration properties. The mechanical interactions between bone and metal lattice have been examined ^14,15^, elucidating the control of lattice anisotropy in bone implant design. Lattice structures have also garnered attention in the aerospace field for structural purposes. Lattice cylindrical panels are employed as shear web attachments^16,17^ in spacecraft primary structures. The feasibility of shell lattice-like structures for spacecraft shielding^18^ have been investigated and compared to traditional shields. Lattices are also examined for aeronautical structural applications. Their potential is harnessed for anti-icing wing systems^19^, leveraging their heat management and energy absorption properties. Notably, the modeling and computational analysis of energy absorption phenomena in lattice structures is an essential topic, considering their exceptional volumetric and specific energy absorption properties^20,21^. Additively manufactured structures with periodic pattern similar to lattice have also been investigated for enhancing properties of CFRP-metal joints^22^.

Due to the increasing interest in utilizing lattices for structural purposes, there is a need to investigate various reliability properties associated with these materials. Fatigue, particularly in regulated fields such as aeronautics and biomedicine, can significantly impact structural reliability. The fatigue properties of lattices have already been studied, either by characterizing the fatigue behavior of parent materials or by applying traditional fatigue analysis methods to different lattice topologies^23–36^. Additionally, computational models have been proposed to analyze fatigue in lattices. In an investigation by Alaimo et al.^37^, an immersed boundary method was used to define the fatigue life of as-built lattices. The finite element (FE) method has been employed as a computational tool to predict fatigue life in lattices^38,39^. Homogenization^40^ has been proposed as an additional tool for this type of analysis, in conjunction with FE models. New numerical models have also been formulated: an example can be found in the study from Burr et al.^41^ where a numerical framework based on a damage accumulation law and progressive failure of lattices is presented. Furthermore, studies have examined how lattice features at the topological or manufacturing levels can affect their fatigue properties. Geometrical and topological singularities, such as thread radius, morphological defects, or surface roughness, can have a significant impact on macro-scale mechanical properties, including fatigue, as demonstrated in literature^42–44^. Other factors affecting fatigue performance have also been investigated, such as load direction^45^, printing direction^46,47^, the presence of coatings^48^, and temperature^49^. For the purpose of comparing the various methodologies proposed in this state of the art, a tabular summary can be found in Table 1.

**Table 1:** comparison of the current state of the art about lattice structure subject to fatigue loading conditions.

References	Phenomenon investigated	Methodology used
^23^	Static and fatigue properties of lattice and cellular structures	Experimental investigation
^24^	Compressive fatigue properties of octet-truss lattice structures	Experimental investigation
^25^	Static compressive and fatigue properties of graded lattice	X-ray CT reconstruction and experimental investigation
^26^	Fatigue behavior and biocompatibility of bioactive tantalum graded lattice structures	Experimental investigation
^27^	Effect of different factors on the fatigue behavior of lattice structures	FEM and experimental data comparison
^28^	Fatigue life prediction of triangular lattice structures	Analytical method and experimental investigation
^29^	Compressive fatigue strength of lattice structures	CT reconstruction, FEM and experimental data comparison
^30^	Fatigue crack propagation of octet-truss lattices for different orientations	FEM and experimental data comparison
^31^	Fatigue properties of novel isosurface strut-based lattice structures	FEM and homogenization
^32^	Fatigue behavior of shell lattice structures	Experimental investigation
^33^	Constant amplitude and random fatigue of schwarz triply periodic minimal surface lattice structures	FEM and experimental investigation
^34^	Fatigue damage in micro-lattice materials	CT-based FEM and experimental data comparison
^35^	Static and fatigue properties of graded lattice	FEM and experimental data comparison
^36^	Fatigue properties of gyroid lattice structures with laterial loading	CT reconstruction and experimental investigation
^37^	Fatigue life estimation of lattice structures	FEM
^38^	Fatigue life estimation of lattice structures	FEM
^39^	Damage modeling of lattice structures subject to LCF	FEM and experimental data comparison
^40^	Fatigue analysis of lattice structures in LCF condition	FEM and homogenization
^41^	Fatigue life estimation of lattice structures	FEM and experimental data comparison
^42^	Influence of node fillet, unit cell size and strut orientation on the fatigue behavior of lattice structures	FEM and experimental data comparison
^43^	Influence of geometrical imperfections and defects on the fatigue behavior of lattice structures	FEM and experimental data comparison
^44^	Optimization of lattice structures for superior fatigue performances	Topological optimization, FEM and experimental data comparison
^45^	Influence of load direction on the fatigue behavior of lattice structures	Analytical method and experimental data comparison
^46^	Influence of node and building orientation on the fatigue behavior of lattice structures	Experimental investigation
^47^	Optimization of lattice structures for superior fatigue performances	Topological optimization, FEM and experimental data comparison
^48^	Influence of coating on the fatigue behavior of lattice structures	Experimental investigation
^49^	Thermal and strain analysis of lattice structures subject to LCF	Experimental investigation

Taking into account the unresolved issues that still affect the understanding and prediction of fatigue strength in lattice structures, this study introduces a fatigue failure model that yields results substantiated by experimental validation in terms of fatigue limit prediction and failure localization within the lattice region. In contrast to other methods, the proposed approach is founded upon the evaluation of homogenized stresses at a large scale and the computation of real three-dimensional stresses at a local scale. This characteristic enables computational efficiency and facilitates the identification of fatigue failure initiation points. The de-homogenization process is employed to access the effective stress distribution at the critical point of the structure. Overall, the model encompasses a few key steps: firstly, the lattice structure is homogenized at the level of each cell, accounting for different sizes and shapes. Secondly, a strain-based formulation is utilized to determine the location of the most heavily loaded cell within the structure, which corresponds to the fracture nucleation point. Finally, the de-homogenization process is applied solely to the critical cell, allowing the retrieval of the real three-dimensional stress distribution at each point within the discretized volume of this cell. Subsequently, a multi-axial fatigue failure criterion is applied to this stress distribution. The model is validated through an experimental campaign involving lattice samples with a cantilever configuration subjected to cyclic bending loads produced through laser-based powder bed fusion of metals (PBF-LB/M).

## Model

**Figure 1:**
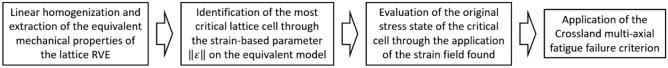
fundamental steps of the model proposed.

The most notable advantage of the proposed method lies in its capacity to determine whether the lattice structure experiences fatigue failure under a given load by analyzing a single critical cell. Through this approach, the method can discern, in relation to a computed fatigue limit value, the survival or failure of the entire lattice structure under fatigue conditions. The proposed model employs various techniques for its implementation, and an overview of the procedural steps, organized according to the utilized technique, is presented in Fig. 1. Subsequently, the model has been applied to a case involving an Inconel 625 cantilever beam composed of octet-truss lattice cells subjected to cyclic bending loads.

Inconel 625 exhibits favorable mechanical properties and finds extensive application in demanding environments, notably within gas turbine applications^50^ and general engine contexts. Employing Inconel 625 lattice structures in such environments has the potential to enhance efficiency and contribute to emissions reduction efforts. The choice of octet-truss cell geometry is motivated by the technological challenges associated with the various orientations of the struts. In this regard, the dominant behavior of the struts is characterized by stretching, a property that imparts greater stiffness to the overall lattice when compared to alternative cell geometries, consequently resulting in an improved strength-to-mass ratio. For the purposes of this study, fully reversed fatigue loading (R = − 1) is adopted, as it affords symmetrical loading conditions and fatigue-induced damage mechanisms for the lattice samples, thereby eliminating result variability stemming from the structure’s orientation.

### Homogenization

The analytical formulation of the model pertains to a generic lattice cell type. The initial step entails the homogenization process, which is commonly employed in micromechanics to derive equivalent material properties of a continuum solid with multiple phases and periodic repetition of a RVE. This technique has been extensively utilized for composite materials and lattices alike. A single lattice cell can be regarded as the RVE, whereby the stiffness matrix associated with the RVE is computed and utilized to define the mechanical properties of the orthotropic equivalent material.

Six static simulations are conducted to assess the components of the stiffness matrix [C] as indicated in Eq. (1), where $\left\{ {\overline{\sigma }} \right\}$ and $\left\{ {\overline{\varepsilon }} \right\}$ represent the average stress and average strain vectors, respectively.

1 $$\left\{ {\begin{array}{*{20}c} {\begin{array}{*{20}c} {\overline{{\sigma _{1} }} } \\ {\overline{{\sigma _{2} }} } \\ \end{array} } \\ {\begin{array}{*{20}c} {\overline{{\sigma _{3} }} } \\ {\overline{{\sigma _{4} }} } \\ \end{array} } \\ {\begin{array}{*{20}c} {\overline{{\sigma _{5} }} } \\ {\overline{{\sigma _{6} }} } \\ \end{array} } \\ \end{array} } \right\} = \left[ {\begin{array}{*{20}c} {C_{{11}} } & \cdots & {C_{{16}} } \\ {C_{{21}} } & \cdots & {C_{{26}} } \\ \vdots & \ddots & \vdots \\ {C_{{61}} } & \cdots & {C_{{66}} } \\ \end{array} } \right]\left\{ {\begin{array}{*{20}c} {\begin{array}{*{20}c} {\overline{{\varepsilon _{1} }} } \\ {\overline{{\varepsilon _{2} }} } \\ \end{array} } \\ {\begin{array}{*{20}c} {\overline{{\varepsilon _{3} }} } \\ {\overline{{\gamma _{4} }} } \\ \end{array} } \\ {\begin{array}{*{20}c} {\overline{{\gamma _{5} }} } \\ {\overline{{\gamma _{6} }} } \\ \end{array} } \\ \end{array} } \right\}$$

The assumption of constant strain energy remains valid as both the actual lattice cell and the homogenized cell experience an equivalent amount of strain energy during deformation. Each of the six simulations to be conducted should exhibit one of the six unitary average strains in the vector $\left\{\overline{\varepsilon }\right\}$. Hence, it can be asserted that the average strain vector components correspond to the RVE strain component (denoted as ${\varepsilon }_{\beta }^{0}$), utilized in the simulations (2). By performing these simulations, it becomes possible to evaluate the six components of the stiffness matrix based on Eq. (3).

2 $$\overline{{\varepsilon_{i} }} = \frac{1}{V}\mathop \smallint \limits_{V} \varepsilon_{i} dV = \varepsilon_{i}^{0}$$

3 $$C_{\alpha \beta } = \overline{{\sigma_{\alpha } }} = \mathop \smallint \nolimits_{V} \sigma_{\alpha } \left( {x_{1} ,x_{2} ,x_{3} } \right)dV \quad where \varepsilon_{\beta }^{0} = 1 \quad for \alpha ,\beta = 1, \ldots , 6$$

Periodic boundary conditions are essential in each simulation, accompanied by the imposition of specific displacements to achieve an RVE strain of 1. The applied displacements on each face of the RVE are outlined in Table 2, where a_1_, a_2_, and a_3_ denote the half edge lengths of the RVE along the x_1_, x_2_, and x_3_ axes of the coordinate system located at the cell's center.

**Table 2:** summary of boundary conditions applied to the RVE for the homogenization process: Ui terms represent the degrees of freedom (DOF) of displacement for the RVE nodes in the (x_1_, x_2_, x_3_) coordinate system

RVE face	1st analysis			2nd analysis			3rd analysis			4th analysis			5th analysis			6th analysis		
	$\overline{{\varepsilon }_{1}}=1$			$\overline{{\varepsilon }_{2}}=1$			$\overline{{\varepsilon }_{3}}=1$			$\overline{{\gamma }_{4}}=1$			$\overline{{\gamma }_{5}}=1$			$\overline{{\gamma }_{6}}=1$		
	***U***_***1***_	***U***_***2***_	***U***_***3***_	***U***_***1***_	***U***_***2***_	***U***_***3***_	***U***_***1***_	***U***_***2***_	***U***_***3***_	***U***_***1***_	***U***_***2***_	***U***_***3***_	***U***_***1***_	***U***_***2***_	***U***_***3***_	***U***_***1***_	***U***_***2***_	***U***_***3***_
${{\varvec{x}}}_{1}={-{\varvec{a}}}_{1}$	${-a}_{1}$	/	/	0	/	/	0	/	/	0	/	/	/	0	${-a}_{1}$	/	$-{a}_{1}$	0
${{\varvec{x}}}_{1}={{\varvec{a}}}_{1}$	${a}_{1}$	/	/	0	/	/	0	/	/	0	/	/	/	0	${a}_{1}$	/	${a}_{1}$	0
${{\varvec{x}}}_{2}={-{\varvec{a}}}_{2}$	/	0	/	/	$-{a}_{2}$	/	/	0	/	0	/	$-{a}_{2}$	/	0	/	${-a}_{2}$	/	0
${{\varvec{x}}}_{2}={{\varvec{a}}}_{2}$	/	0	/	/	${a}_{2}$	/	/	0	/	0	/	${a}_{2}$	/	0	/	${a}_{2}$	/	0
${{\varvec{x}}}_{3}={-{\varvec{a}}}_{3}$	/	/	0	/	/	0	/	/	$-{a}_{3}$	0	$-{a}_{3}$	/	$-{a}_{3}$	0	/	/	/	0
${{\varvec{x}}}_{3}={{\varvec{a}}}_{3}$	/	/	0	/	/	0	/	/	${a}_{3}$	0	${a}_{3}$	/	${a}_{3}$	0	/	/	/	0

"0" denotes constrained DOF, "/" denotes free DOF.

The strain components of the RVE, as defined in Table 2, can also be described by Eqs. (4).

4 $$\begin{gathered} \left\{ {\begin{array}{*{20}c} {u_{1} \left( {a_{1} ,x_{2} ,x_{3} } \right) - u_{1} \left( { - a_{1} ,x_{2} ,x_{3} } \right) = 2a_{1} \varepsilon_{11}^{0} } \\ {u_{2} \left( {a_{1} ,x_{2} ,x_{3} } \right) - u_{2} \left( { - a_{1} ,x_{2} ,x_{3} } \right) = 2a_{1} \varepsilon_{21}^{0} } \\ {u_{3} \left( {a_{1} ,x_{2} ,x_{3} } \right) - u_{3} \left( { - a_{1} ,x_{2} ,x_{3} } \right) = 2a_{1} \varepsilon_{31}^{0} } \\ \end{array} } \right. \hfill \\ \left\{ {\begin{array}{*{20}c} {u_{1} \left( {x_{1} ,a_{2} ,x_{3} } \right) - u_{1} \left( {x_{1} , - a_{2} ,x_{3} } \right) = 2a_{2} \varepsilon_{12}^{0} } \\ {u_{2} \left( {x_{1} ,a_{2} ,x_{3} } \right) - u_{2} \left( {x_{1} , - a_{2} ,x_{3} } \right) = 2a_{2} \varepsilon_{22}^{0} } \\ {u_{3} \left( {x_{1} ,a_{2} ,x_{3} } \right) - u_{3} \left( {x_{1} , - a_{2} ,x_{3} } \right) = 2a_{2} \varepsilon_{32}^{0} } \\ \end{array} } \right. \hfill \\ \left\{ {\begin{array}{*{20}c} {u_{1} \left( {x_{1} ,x_{2} ,a_{3} } \right) - u_{1} \left( {x_{1} ,x_{2} , - a_{3} } \right) = 2a_{3} \varepsilon_{13}^{0} } \\ {u_{2} \left( {x,x_{2} ,a_{3} } \right) - u_{2} \left( {x_{1} ,x_{2} , - a_{3} } \right) = 2a_{3} \varepsilon_{23}^{0} } \\ {u_{3} \left( {x_{1} ,x_{2} ,a_{3} } \right) - u_{3} \left( {x_{1} ,x_{2} , - a_{3} } \right) = 2a_{3} \varepsilon_{33}^{0} } \\ \end{array} } \right. \hfill \\ \end{gathered}$$

The edges require their own set of conditions since each edge belongs to two faces of the RVE simultaneously. Their respective equations are defined in (5).

5 $$\begin{gathered} \left\{ {\begin{array}{*{20}c} {u_{i} \left( {a_{1} ,a_{2} ,x_{3} } \right) - u_{i} \left( { - a_{1} , - a_{2} ,x_{3} } \right) = 2a_{1} \varepsilon_{i1}^{0} + 2a_{2} \varepsilon_{i2}^{0} } \\ {u_{i} \left( {a_{1} , - a_{2} ,x_{3} } \right) - u_{i} \left( { - a_{1} ,a_{2} ,x_{3} } \right) = 2a_{1} \varepsilon_{i1}^{0} - 2a_{2} \varepsilon_{i2}^{0} } \\ \end{array} \quad with \;i = 1,2,3} \right. \hfill \\ \left\{ {\begin{array}{*{20}c} {u_{i} \left( {a_{1} ,x_{2} ,a_{3} } \right) - u_{i} \left( { - a_{1} ,x_{2} , - a_{3} } \right) = 2a_{1} \varepsilon_{i1}^{0} + 2a_{3} \varepsilon_{i3}^{0} } \\ {u_{i} \left( {a_{1} ,x_{2} , - a_{3} } \right) - u_{i} \left( { - a_{1} ,x_{2} ,a_{3} } \right) = 2a_{1} \varepsilon_{i1}^{0} - 2a_{3} \varepsilon_{i3}^{0} } \\ \end{array} \quad with \;i = 1,2,3} \right. \hfill \\ \left\{ {\begin{array}{*{20}c} {u_{i} \left( {x_{1} ,a_{2} ,a_{3} } \right) - u_{i} \left( {x_{1} , - a_{2} , - a_{3} } \right) = 2a_{2} \varepsilon_{i2}^{0} + 2a_{3} \varepsilon_{i3}^{0} } \\ {u_{i} \left( {x_{1} ,a_{2} , - a_{3} } \right) - u_{i} \left( {x_{1} , - a_{2} ,a_{3} } \right) = 22\varepsilon_{i2}^{0} - 2a_{3} \varepsilon_{i3}^{0} } \\ \end{array}\quad with \;i = 1,2,3} \right. \hfill \\ \end{gathered}$$

Similar considerations apply to the corners of the RVE, for which a specific set of constraints has also been defined (6).

6 $$\left\{ {\;\begin{array}{*{20}c} {u_{i} \left( {a_{1} ,a_{2} ,a_{3} } \right) - u_{i} \left( { - a_{1} , - a_{2} , - a_{3} } \right) = 2a_{1} \varepsilon_{i1}^{0} + 2a_{2} \varepsilon_{i2}^{0} + 2a_{3} \varepsilon_{i3}^{0} } \\ {u_{i} \left( {a_{1} ,a_{2} , - a_{3} } \right) - u_{i} \left( { - a_{1} , - a_{2} ,a_{3} } \right) = 2a_{1} \varepsilon_{i1}^{0} + 2a_{2} \varepsilon_{i2}^{0} - 2a_{3} \varepsilon_{i3}^{0} } \\ {u_{i} \left( { - a_{1} ,a_{2} ,a_{3} } \right) - u_{i} \left( {a_{1} , - a_{2} , - a_{3} } \right) = - 2a_{1} \varepsilon_{i1}^{0} + 2a_{2} \varepsilon_{i2}^{0} + 2a_{3} \varepsilon_{i3}^{0} } \\ {u_{i} \left( {a_{1} , - a_{2} ,a_{3} } \right) - u_{i} \left( { - a_{1} ,a_{2} , - a_{3} } \right) = 2a_{1} \varepsilon_{i1}^{0} - 2a_{2} \varepsilon_{i2}^{0} + 2a_{3} \varepsilon_{i3}^{0} } \\ \end{array} \;\; with\; i = 1,2,3\;} \right.$$

Once the stiffness matrix is defined using Eq. (3), the compliance matrix is calculated using Eq. (7). Finally, Eqs. (8), (9), and (10) outline the procedure for evaluating the equivalent orthotropic mechanical properties of the material.

7 $$\left[ S \right] = \left[ C \right]^{ - 1}$$

8 $$E_{1} = \frac{1}{{S_{11} }} \quad E_{2} = \frac{1}{{S_{22} }} \quad E_{3} = \frac{1}{{S_{33} }}$$

9 $$G_{23} = \frac{1}{{S_{44} }} \quad G_{13} = \frac{1}{{S_{55} }} \quad G_{12} = \frac{1}{{S_{66} }}$$

10 $$\nu_{12} = - S_{12} \cdot E_{1} \quad \nu_{23} = - S_{23} \cdot E_{2} \quad \nu_{13} = - S_{13} \cdot E_{1}$$

### Critical cell identification

By utilizing these properties, the lattice section of the analyzed sample or component can be modeled as a bulk equivalent material, resulting in a computationally efficient and streamlined FEM model.

To conduct the fatigue analysis, the mean load and alternate load are separated and treated as static loads in a FEM simulation. The primary output of these simulations is the norm of the strain tensor, as defined in Eq. (11), which is evaluated for each element of the equivalent material representing the lattice. Each element corresponds to a single lattice cell. The components of the strain tensor, outlined in Eq. (12), are saved as they will be utilized in the subsequent de-homogenization process.

11 $$\varepsilon = \sqrt {\mathop \sum \limits_{i} \mathop \sum \limits_{j} \varepsilon_{ij} \cdot \varepsilon_{ij} }$$

12 $$\left[ \varepsilon \right] = \left[ {\begin{array}{*{20}c} {\varepsilon_{xx} } & {\varepsilon_{xy} } & {\varepsilon_{xz} } \\ {\varepsilon_{yx} } & {\varepsilon_{yy} } & {\varepsilon_{yz} } \\ {\varepsilon_{zx} } & {\varepsilon_{zy} } & {\varepsilon_{zz} } \\ \end{array} } \right]$$

The most heavily loaded (or critical) cell within the homogenized material is determined based on the maximum $\Vert \varepsilon \Vert$ value. Assuming this cell represents the most loaded region of the lattice structure, it is presumed to possess the lowest fatigue life compared to other cells. Consequently, the fracture nucleation is expected to initiate from this specific location. Consequently, the subsequent calculations concentrate solely on this critical cell.

### De-homogenization and fatigue limit estimation

The subsequent step in the method involves the de-homogenization of the critical cell. The stored strain tensor containing the strain components of the critical cell is utilized. A physically accurate static model of the RVE with the actual lattice shape is constructed, and the aforementioned strain field (12) is applied to retrieve the actual stress distribution within the lattice cell as the primary output.

Subsequently, the most heavily loaded point of the structure is identified, and the multi-axial stress components of the critical cell are computed based on the nominal mean and alternate external load components, respectively. At this stage, one of the multi-axial fatigue methods can be employed to estimate the fatigue life of the structure by analyzing its critical point. The Crossland criterion^51^ is one such method suitable for this investigation and is subsequently applied. However, it may be challenging to determine the fatigue limit necessary for the application of the Crossland method for certain materials. Additionally, this criterion has limitations regarding the representation of actual load combinations, as discussed by Navarro et al.^52^. For instance, it does not account for the mixed effects of static and alternate loads. Nevertheless, the primary advantage of the Crossland criterion lies in its simplicity of application and calculation.

The application of the Crossland multi-axial fatigue criterion is predicated on the assessment of an equivalent stress (13), derived from the principal stresses along the three axes. Apart from its simplicity for computational analysis across various loading scenarios, such as bending and torsion, the Crossland criterion is also a conventional approach applicable to high cycle fatigue (HCF)^53^. Furthermore, numerous instances of the Crossland criterion's application to nickel-based superalloy samples can be found in the literature. Notably, examples wherein this criterion was employed for the fatigue failure analysis of Inconel 718 samples and gas turbine blades can be found in literature^54,55^. In order to ensure infinite life, the Crossland equivalent stress must be lower than the fatigue torsional limit ${\tau }_{f}$.

13 $$\tau_{CROSS} = \sqrt {J_{2,a} } + \left( {\frac{{3\tau_{f} }}{{\sigma_{f} }} - \sqrt 3 } \right)\sigma_{H,max} \le \tau_{f}$$

The Crossland stress is computed using the fatigue limits for shear and bending ($\sigma_{f}$), the second invariant of the deviatoric stress tensor $J_{2,a}$ as defined in (14), and the maximum hydrostatic stress $\sigma_{H,max}$ obtained from expression (15).

14 $$\sqrt {J_{2,a} } = \frac{{\sigma_{eq} }}{\sqrt 3 }$$

15 $$\sigma_{H,max} = \frac{{S_{1} + S_{2} + S_{3} }}{3}$$

Within expressions (14) and (15), $\sigma_{eq}$ represents the Von Mises equivalent stress, while $S_{1}$, $S_{2}$, and $S_{3}$ denote the principal stresses along the three principal directions. A viable alternative, often employed as a multi-axial criterion in such cases, is the Sines criterion^56^, wherein the maximum hydrostatic stress is replaced by the mean stress while maintaining the formulation of Eq. (13) unchanged.

## Modeling of cantilever lattice beam with bending load

**Figure 2:**
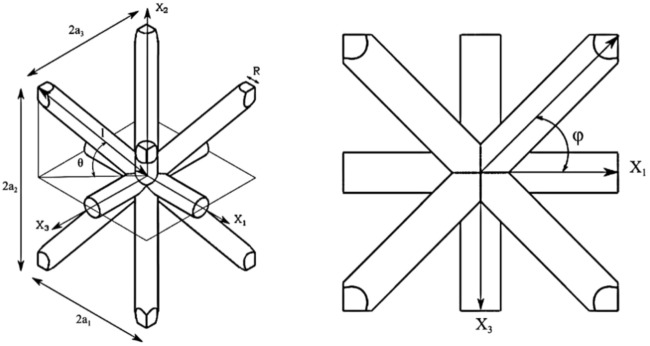
octa-truss lattice cell geometry and dimensions.

The previously introduced model is implemented for a cantilever beam subjected to cyclic bending load. The octa-cell lattice cell, as shown in Fig. 2, is employed in this analysis. Both uniform and graded lattice cell configurations are considered for the samples. Referring to the labels in the accompanying figure, the nominal dimensions of the lattice cell are *l* = 2 mm, *θ* = *φ* = 45°.

The strut diameter is 0.5 mm, while the relationships between the cell edge dimensions are given by $a_{1}=a_{3}=l/2$ and ${a}_{2}=l\bullet sin\theta$. These cell dimensions apply to both the uniform lattice and the graded lattice, with the only variation being the strut radius. In the case of graded lattices, the strut diameters range from 0.5 mm (uniform) to 1.1 mm (graded).

**Figure 3:**
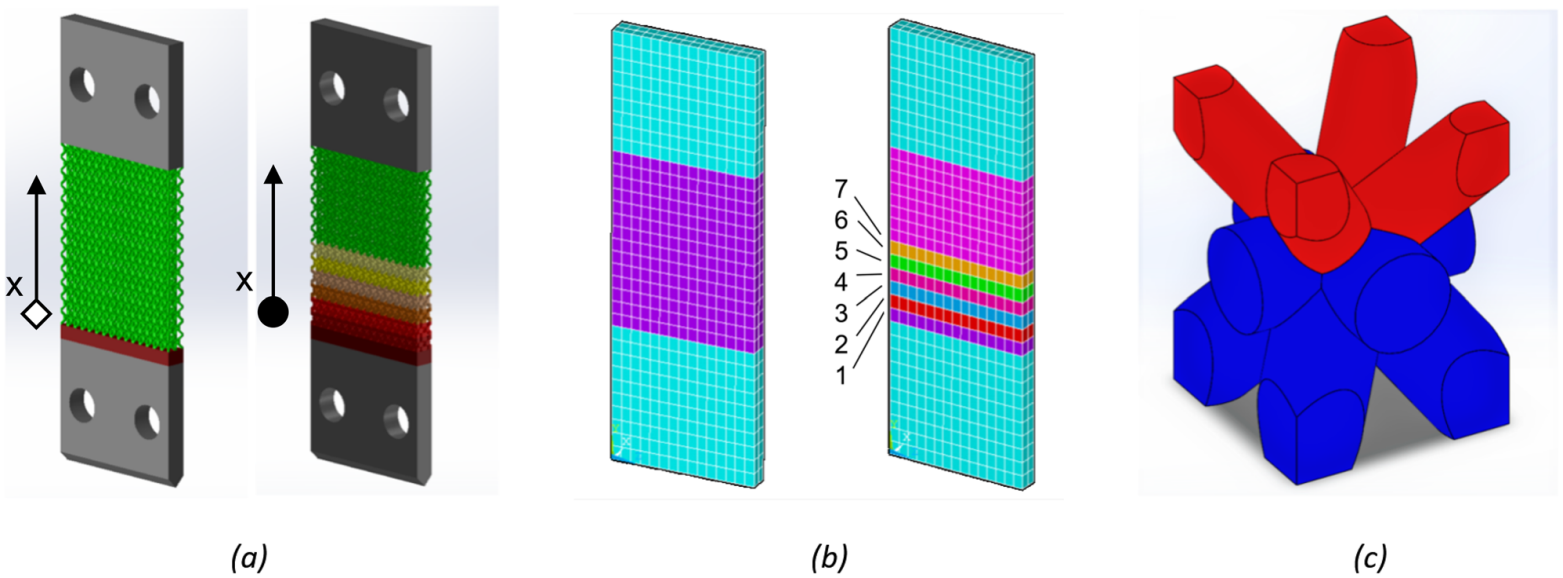
samples geometry with uniform and graded lattice (**a**). Homogenized models where colors different from light blue (that is bulk material) represent homogenized materials; for the graded sample (right), colors ranging from purple (bottom) to pink (top) represent cells where the diameter decreases from 1.1 mm on row 1 to 0.5 mm on row 7 (**b**). Example of a single cell for the graded sample, where the diameter switch occurs in the middle of the cell to ensure continuity at the nodes (**c**).

The sample has overall dimensions of 90 mm in length, 30 mm in width, and 4 mm in thickness. The lattice region has a length of 36.7 mm (Fig. 3a, left), while the bulk region serves the purpose of securing the sample to the testing system. In the case of the graded sample version (Fig. 3a, right), cell grading is implemented in the lower half of the lattice region. A mesh sensitivity analysis, relying on equivalent stress, was conducted on the lattice RVE prior to implementing the homogenization process. This analysis determined an optimal mesh size of 0.04 mm. Following the homogenization process, the material's equivalent properties are differentiated based on the qualitative distribution depicted in Fig. 3b. The graded region of the lattice is designed to ensure structural continuity among rows of cells. This is achieved through the cell configuration illustrated in Fig. 3c, where the strut diameter varies at the central node between the lower and upper parts of the cell. The graded region consists of seven rows of cells (Fig. 3c) identified by two different diameter values (lower and upper parts). The seventh row of cells is repeated uniformly until the end of the lattice region. For simulations involving the homogenized material, the mesh size was set at 2 mm for both the thickness and transversal dimensions. Additionally, a mesh seed was placed within the lattice region along the longitudinal dimension. These dimensions were chosen to ensure alignment between the volumes of the RVE and the homogenized element.

The lattice and bulk material used in this study is Inconel-625, characterized by a Young's modulus of *E* = 250 GPa, a Poisson's ratio of υ = 0.308, and a shear modulus of *G* = 78.364 GPa. The fatigue limit of this material, calculated with the Crossland criterion, are obtained from a previous investigation^57^:

$$\sigma_{f} = 240\; MPa\quad\; \tau_{f} = 0,577\cdot \sigma_{f} = 138.48 \;MPa$$

The sample is subjected to cyclic loading, where the static component is zero, meaning no applied preload. The variable load follows a sinusoidal pattern with a constant amplitude (R = σ_min_/σ_max_ = − 1). For modeling purposes, only the information about the maximum (or minimum) applied load is required, which has been determined as 100 N. In the homogenized FEM model, a 100 N load is distributed among all the nodes at one end of the sample, while the other end is completely fixed.

## Experimental validation

The described experimental campaign aims to validate the fatigue failure predicted by the model. Samples are produced using the AM process called laser-based powder bed fusion of metals (PBF-LB/M), employing Inconel 625 material with identical geometrical properties and dimensions as the structures analyzed in the model. The actual sample dimensions, including process tolerances, are taken into account. The Renishaw AM 500 M machine was employed for the manufacturing of the samples. These samples were fabricated in the vertical direction, eliminating the necessity for supports and consequently avoiding issues associated with support removal. They have not undergone any thermal or mechanical processing and are tested in their as-built condition.

**Figure 4:**
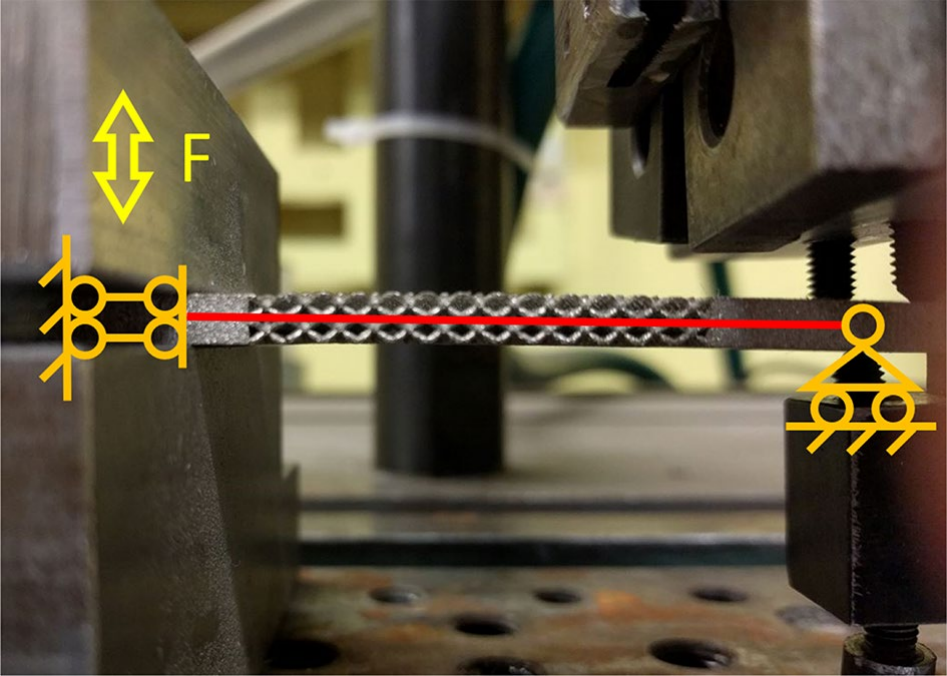
experimental test with the schematization of the fixtures used. On the left side, the sample was clamped into the moving head of the machine, permitting only vertical translation. Conversely, at the opposite end, the sample was secured in place using screws, enabling rotation while preventing translations.

**Figure 5:**
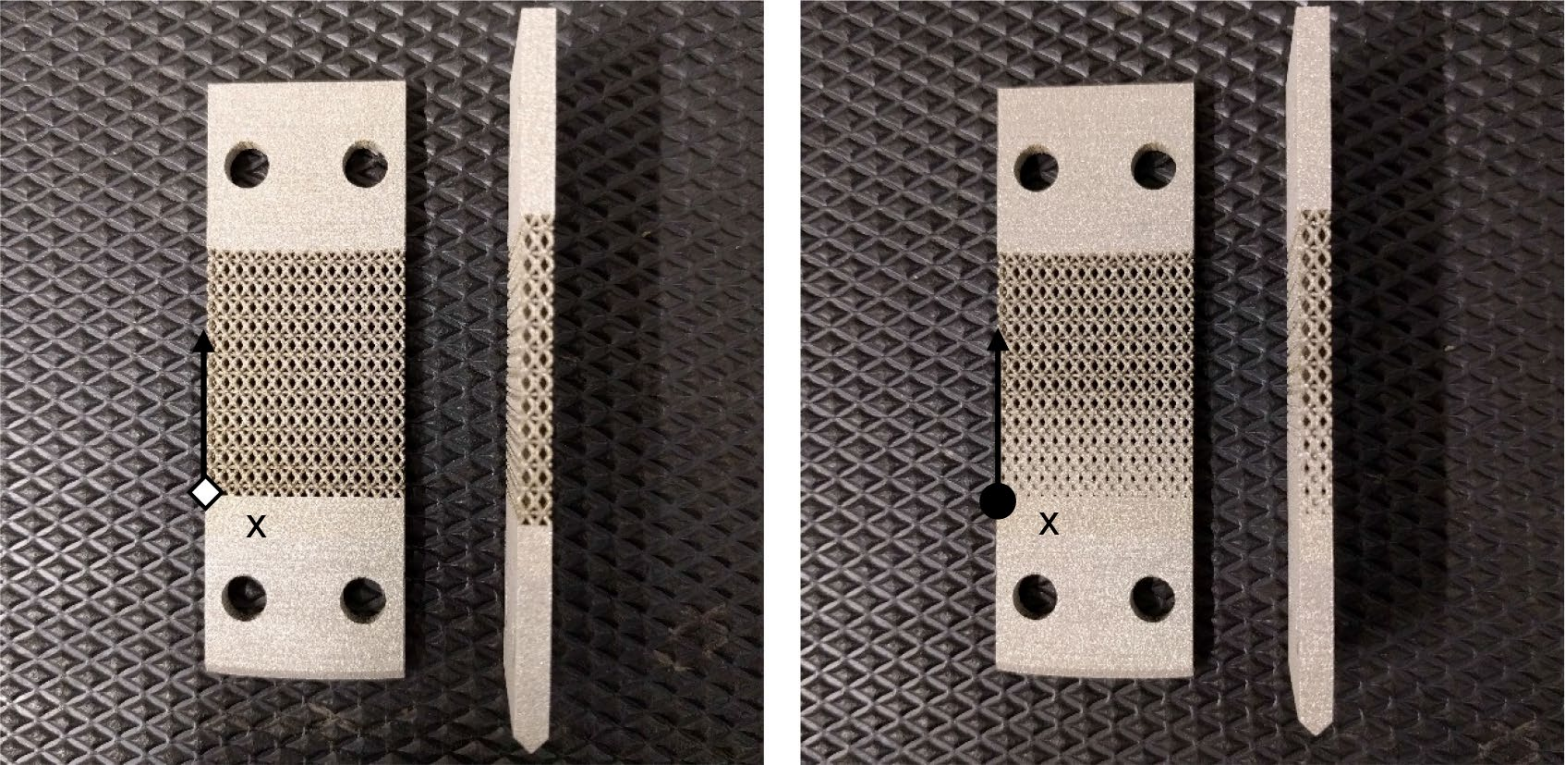
uniform (left) and graded (right) lattice samples produced via additive manufacturing with L-PBF.

For the testing, the Baldwin SF-01-U vibrating system is utilized. This machine enables the application of static load by adjusting the lower head, while the alternate load is generated through the excitation of an eccentric electric rotor. The machine works at constant frequency of 30 Hz. Special grips, as depicted in Fig. 4, have been designed to ensure pure bending load for the samples. The left end of the sample is fully secured within the grips connected to the moving head of the machine. The right end is constrained along the vertical load direction, allowing rotation of the transverse section of the sample through single-point contacts. Furthermore, these grips facilitate bending in both directions, enabling a loading condition with $R=-1$. A total of nine samples, comprising uniform and graded configurations (Fig. 5), have been manufactured and subjected to testing under various load amplitudes ranging from 50 to 200 N, with no preload (static load equal to zero). The sample's particular geometry and lattice cell topology feature a large thermal exchange surface, facilitating efficient thermal dissipation into the surrounding air. As a result, the samples do not undergo self-heating during the tests.

## Results and discussions

### Numerical results

In this section, we present the comparison between the results obtained from the model and the experimental tests conducted on the cantilever samples.

The homogenization process is applied to both the uniform and graded lattice cells. Referring to the cell grading illustrated in Fig. 3, the calculated equivalent properties of the RVEs are presented in Table 3. The cells are numbered in the same manner as in Fig. 3b, and the respective strut diameter values for the upper and lower parts of each cell are provided. The values of Young's modulus (*E*_i_) and shear modulus (*G*_i_) demonstrate the relationship between the strut diameters (or cell density) and the mechanical strength. This distribution of structural properties within the sample plays a crucial role in the failure localization, as described in the subsequent analysis.

**Table 3:** equivalent mechanical properties of the lattice cells after the homogenization process.

RVE	Cell 1	Cell 2	Cell 3	Cell 4	Cell 5	Cell 6	Cell 7 and beyond
Upper/lower diameter [mm]	0.5/0.5 (uniform)	0.5/0.6	0.6/0.7	0.7/0.8	0.8/0.9	0.9/1	1/1.1
${\varvec{E}}_{1}$ [GPa]	11.092	16.631	26.226	39.968	58.643	82.057	108.527
${\varvec{E}}_{2}$ [GPa]	11.946	16.257	26.344	40.575	59.344	82.304	108.025
${\varvec{E}}_{3}$ [GPa]	11.092	16.627	26.209	39.943	58.607	82.163	108.514
${\varvec{G}}_{12}$ [GPa]	8.343	10.178	14.935	20.874	27.912	35.962	44.800
${\varvec{G}}_{13}$ [GPa]	5.283	7.165	11.344	17.102	24.639	33.853	43.913
${\varvec{G}}_{23}$ [GPa]	8.343	10.178	14.947	20.862	27.929	35.979	44.603
${\varvec{\nu}}_{12}$	0.404	0.385	0.352	0.324	0.300	0.284	0.277
${\varvec{\nu}}_{13}$	0.103	0.120	0.144	0.167	0.192	0.219	0.248
${\varvec{\nu}}_{23}$	0.435	0.376	0.354	0.329	0.304	0.285	0.276

**Figure 6:**
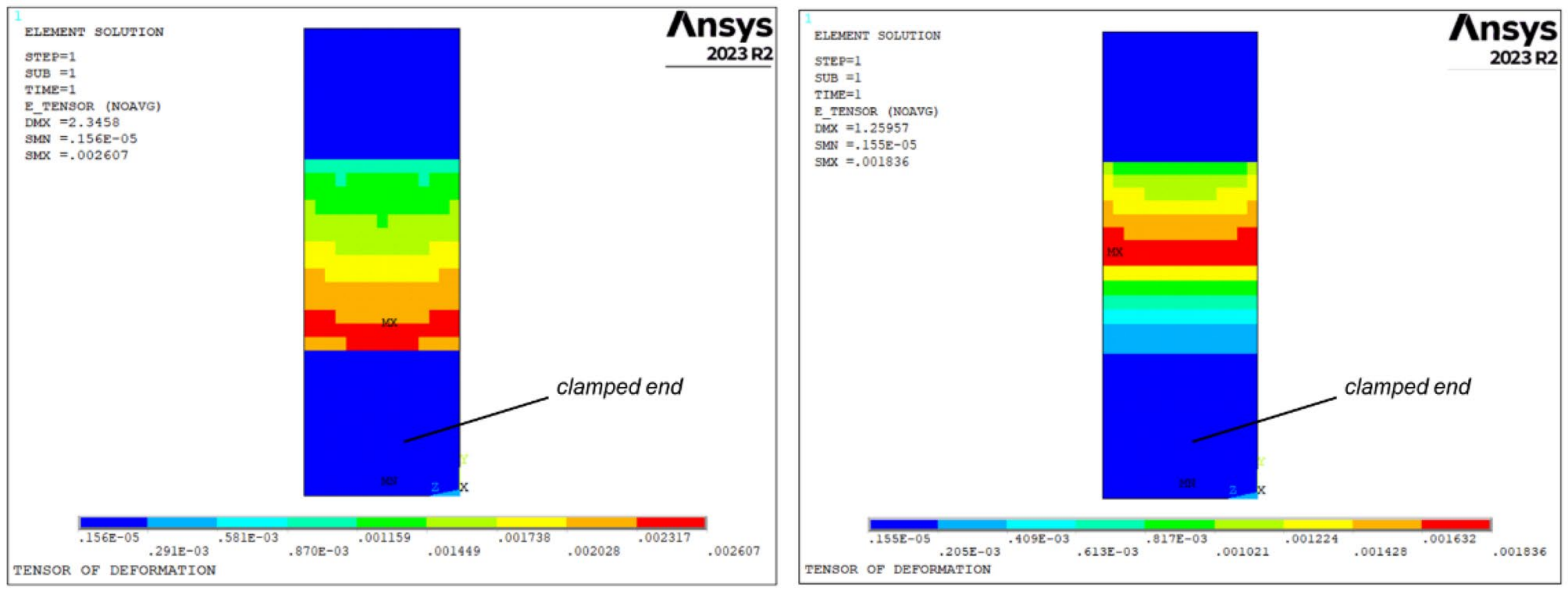
strain tensor norm for the uniform (left) and graded (right) samples after static analysis under the alternate load component (100 N).

The homogenization process is applied to both samples using the aforementioned calculations. In this step, the lattice structures are replaced with equivalent bulk materials that possess the properties listed in Table 3. Subsequently, the norm of the strain tensor is computed for all the equivalent elements, resulting in the plotted results shown in Fig. 6 left (uniform lattice sample) and right (graded lattice sample) when subjected to an alternate bending force of 100 N. As per the methodology's assumption, the region where the initial fracture of the sample is expected to originate is characterized by higher values of the strain tensor norm. In both sample types, this region is highlighted in red. For the uniform lattice sample, the initiation of fracture is near the clamped end where rotation is constrained. However, in the graded lattice sample, the potential region for failure initiation shifts towards the opposite end (where rotation is unrestricted) due to the varying density of cells. Consequently, the lattice grading alters the strain distribution among cells and the location of the failure region.

As previously indicated, the methodology is designed to forecast whether the lattice structure fails when subjected to a specified fatigue load by focusing exclusively on the most critical cell. In essence, to predict fatigue failure, it suffices to examine the stress distribution within the critical region where the norm of the strain tensor is at its maximum. Thus, there is no necessity for conducting extensive and computationally expensive calculations across the entire lattice.

For this purpose, in accordance with the schematic representation in Fig. 1, following the de-homogenization process of the most critical cell, its original shape is recovered, preserving the actual stress distribution within the volume of the struts. Typically, this stress distribution is three-dimensional, and a conventional method for estimating uniaxial equivalent stress can be applied. Various methods are available to achieve this objective, and in the present analysis, the Crossland method is employed for the reasons expounded in Sect. "Model". Moreover, it proves to be more dependable when dealing with fully reversed loads (R = − 1) compared to other methods that rely on the mean stress value, which is zero in this particular case.

The application of the Crossland criterion to the critical cell yields equivalent stress distribution diagrams, as illustrated in Fig. 6 for the uniform lattice sample (right) and the graded lattice sample (left). In both instances, the maximum stress is situated in close proximity to the central node of the cell. Subsequently, the maximum value of the equivalent Crossland stress is compared with the material's fatigue limit to assess the potential occurrence of fatigue failure.

**Figure 7:**
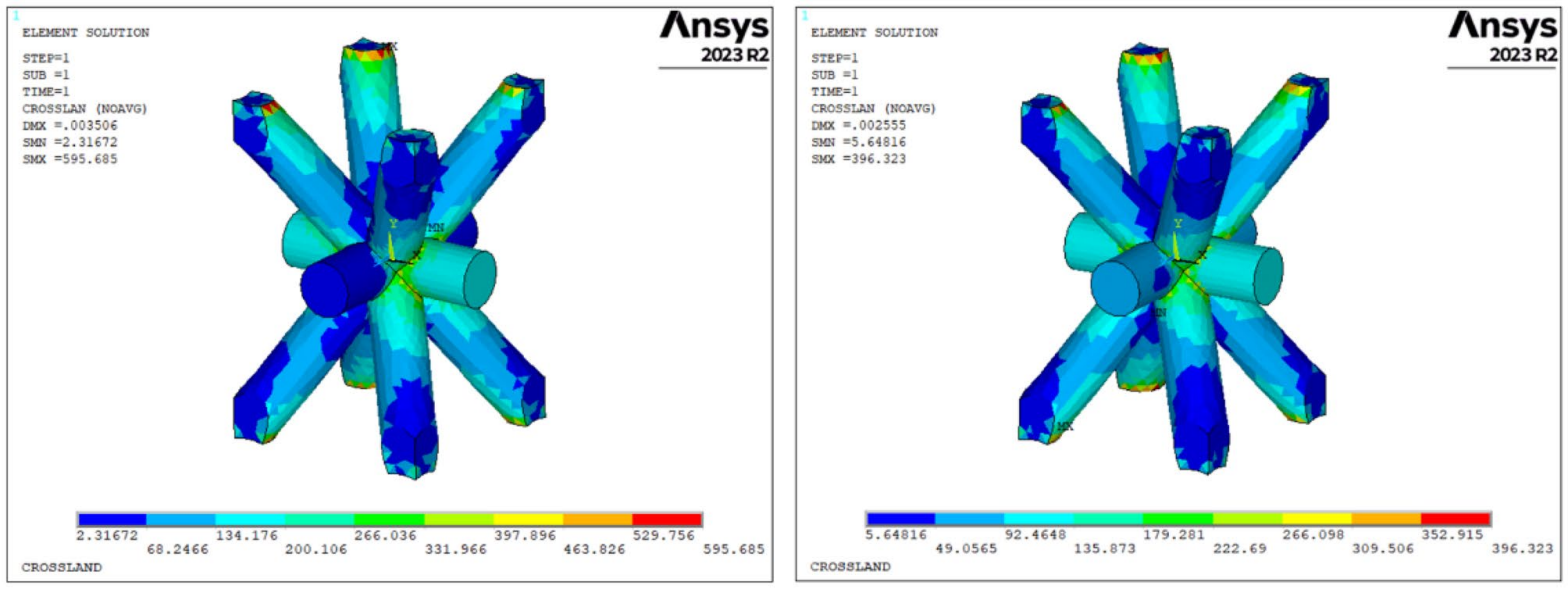
Crossland equivalent stress contour of the most critical cell for the uniform (left) and graded (right) samples, under an applied force of 100 N.

**Figure 8:**
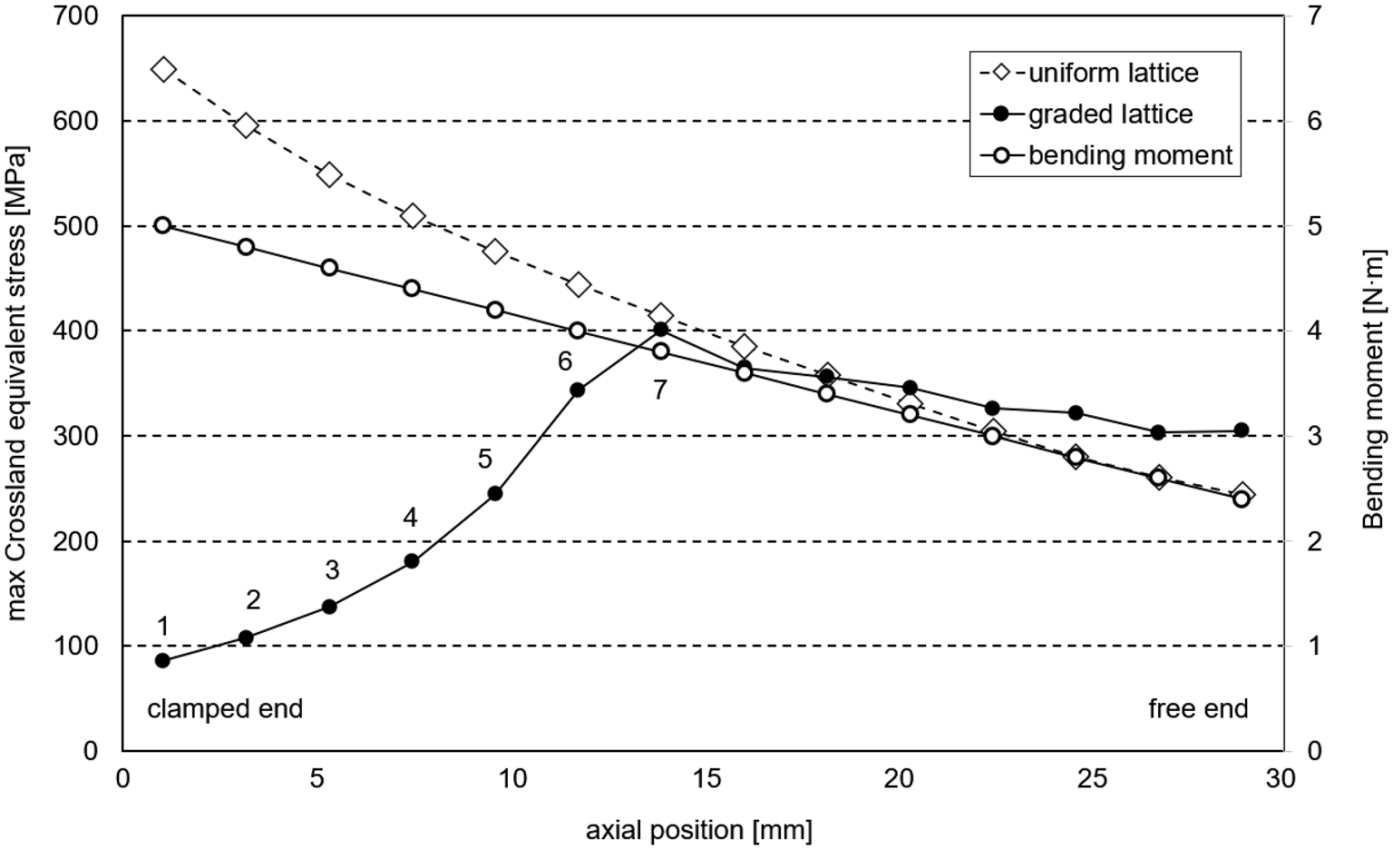
maximum equivalent Crossland stress for the central cell of each row in the uniform and graded lattice samples under an applied force of 100 N and bending moment diagram. The numbers from 1 to 7 indicate the row of cells in the graded lattice sample, with reference to Fig. 3b.

For a more comprehensive insight, the computation of the equivalent Crossland stress is reiterated on a single cell from each row within both the uniform and graded lattice specimens (Fig. 7). Each row comprises cells with distinct strut diameters and varying levels of local bending moments as represented in Fig. 8. The maximum values of equivalent Crossland stress, computed for each cell row, are also illustrated in Fig. 8.

In the uniform lattice sample, as anticipated, the cell row with the highest Crossland stress is row number 1, positioned near the clamped end where the bending moment reaches its maximum. Given the absence of geometric variations among cells, the disparity in equivalent stress among the rows is solely attributable to local loading conditions. The maximum equivalent stress computed in row 1 is ${\tau }_{cross,uniform}^{max}=595.7\text{ MPa}$. A comparative evaluation against the fatigue limit (${\tau }_{f}=138.48 MPa$) reveals that ${\tau }_{cross,uniform}^{max}>{\tau }_{f}$, thereby prognosticating fatigue failure of the lattice structure under the applied load.

A similar procedure can be applied to the graded lattice sample, where the equivalent stress ${\tau }_{cross,graded}^{row1}=92.4\text{ MPa}$ has been identified in row number 1. This value is significantly lower than the fatigue limit, indicating that the influence of cell geometry mitigates the risk of fatigue failure at this specific location within the sample. In row number 1, the higher cell density contributes to a reduction in local stress, and a similar effect is observed in the other rows within the graded region of the sample. Cell density and local loading exert opposing effects on fatigue failure: as we move away from the constrained end, a decrease in cell density favors failure, while a decrease in loading hinders it. Consequently, it is essential to determine whether the combined conditions lead to fatigue failure at a specific point and, if so, pinpoint the location of this failure. Figure 8 illustrates that, in the graded sample, rows 1 through 6 exhibit equivalent stresses lower than those in the corresponding rows of the uniform sample. This outcome is attributed to the thicker struts present in these cells within the graded version. Moving from row number 7 towards the opposite end, the equivalent stresses in both sample types become comparable, with minor variations stemming solely from differences in lattice stiffness, resulting in localized strain variations. The highest value of Crossland equivalent stress is observed in cells within row 7, with ${\tau }_{cross,graded}^{max}=396.3\text{ MPa}$.

In accordance with the model's predictions, fatigue failure is anticipated for both the uniform and lattice samples. The former is projected to fail at row number 1, while the latter is expected to experience failure at row number 7. These locations correspond to the points of maximum equivalent stress and maximum strain tensor norm.

### Experimental results

**Figure 9:**
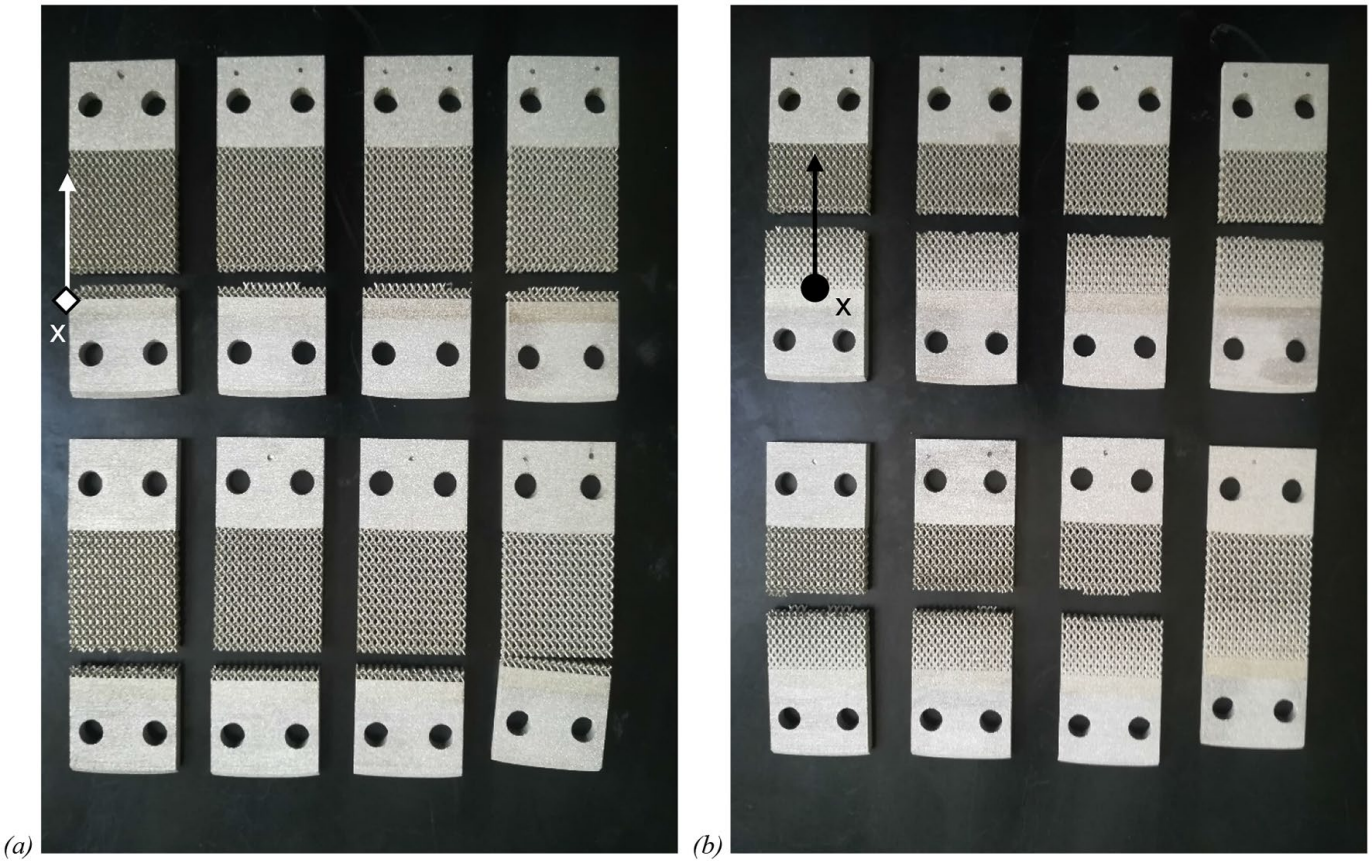
uniform lattice samples (**a**) and graded lattice samples (**b**) after the fatigue tests with evidence of fracture region.

The uniform and lattice samples were subjected to testing in the previously described configuration under a fatigue force of 100 N with R = − 1. The regions of fatigue fracture in all tested samples are depicted in Fig. 9. Notably, the location of the fatigue fracture aligns with the model's predictions. Specifically, the uniform lattice samples exhibit fatigue fractures near row number 1, close to the clamped end, while the graded lattice samples display fractures near row number 7. The highest Crossland stress value in the uniform samples, as depicted in Fig. 8, is situated within the central cell of row 1. Nevertheless, the average stress value across the entire row is greater in row 2. Consequently, the observed experimental fracture location exhibits a slight deviation from the lattice border. Only one out of eighteen samples did not exhibit fatigue failure. The experimental results validate the predicted failure locations by the model, affirming that fractures occur at the cell (or cells within the row, in the case of the current samples) characterized by the maximum strain tensor norm.

**Figure 10:**
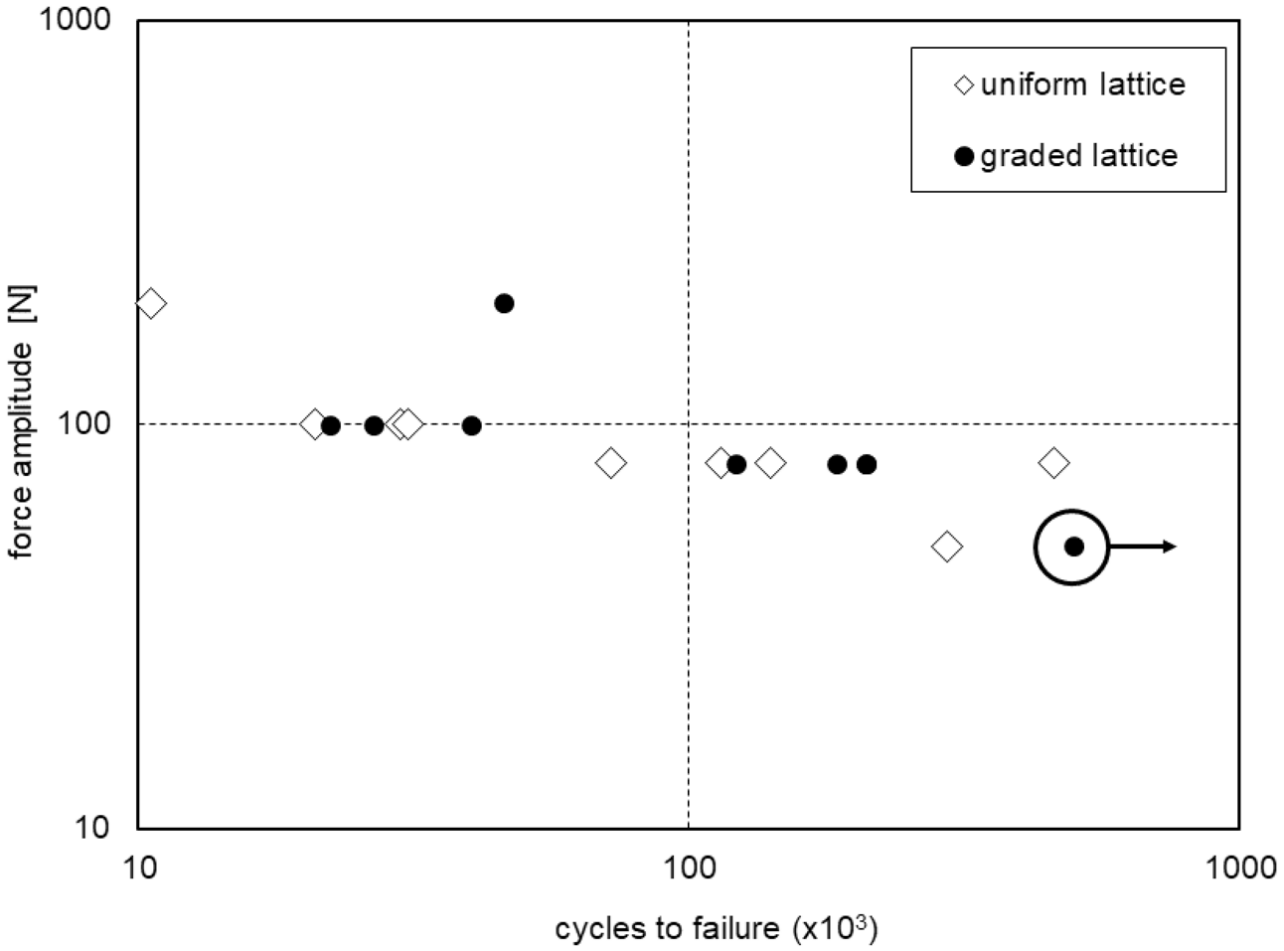
relationship between force amplitude and number of cycles to failure for experimental tests in logarithmic scale.

The experimental results, specifically the load and the number of cycles to failure (S–N curve), are presented in Fig. 10 and Table 4. These findings do not reveal significant disparities in terms of the fatigue lifetime between the two sample typologies. This lack of distinction can likely be attributed to the small difference in the applied load between rows 1 and 7 within this specific sample configuration, which theoretically amounts to approximately 22% in terms of bending moment, as illustrated in Fig. 8. The methodology excels in predicting the position of fatigue fracture, which is contingent upon the maximum strain tensor norm and influenced by cell geometry. These same properties contribute to the limited differences in the actual local stress applied. Additionally, the presence of experimental uncertainties associated with fatigue results necessitates a larger sample population for the construction of S–N curves. In summary, the experimental results do not corroborate the anticipated variation in fatigue lifetime between the sample types.

**Table 4:** number of cycles to failure and relative alternate bending load for tested samples.

Uniform lattice samples		Graded lattice samples	
Number of cycles to failure	Alternate bending load [N]	Number of cycles to failure	Alternate bending load [N]
10,537	200	45,881	200
29,875	100	40,085	100
30,941	100	22,277	100
20,965	100	26,740	100
140,414	80	121,221	80
72,036	80	209,159	80
114,402	80	185,480	80
459,770	80	209,238	80
294,000	50	(not failed)	50

### Discussions about fatigue behavior

**Figure 11:**
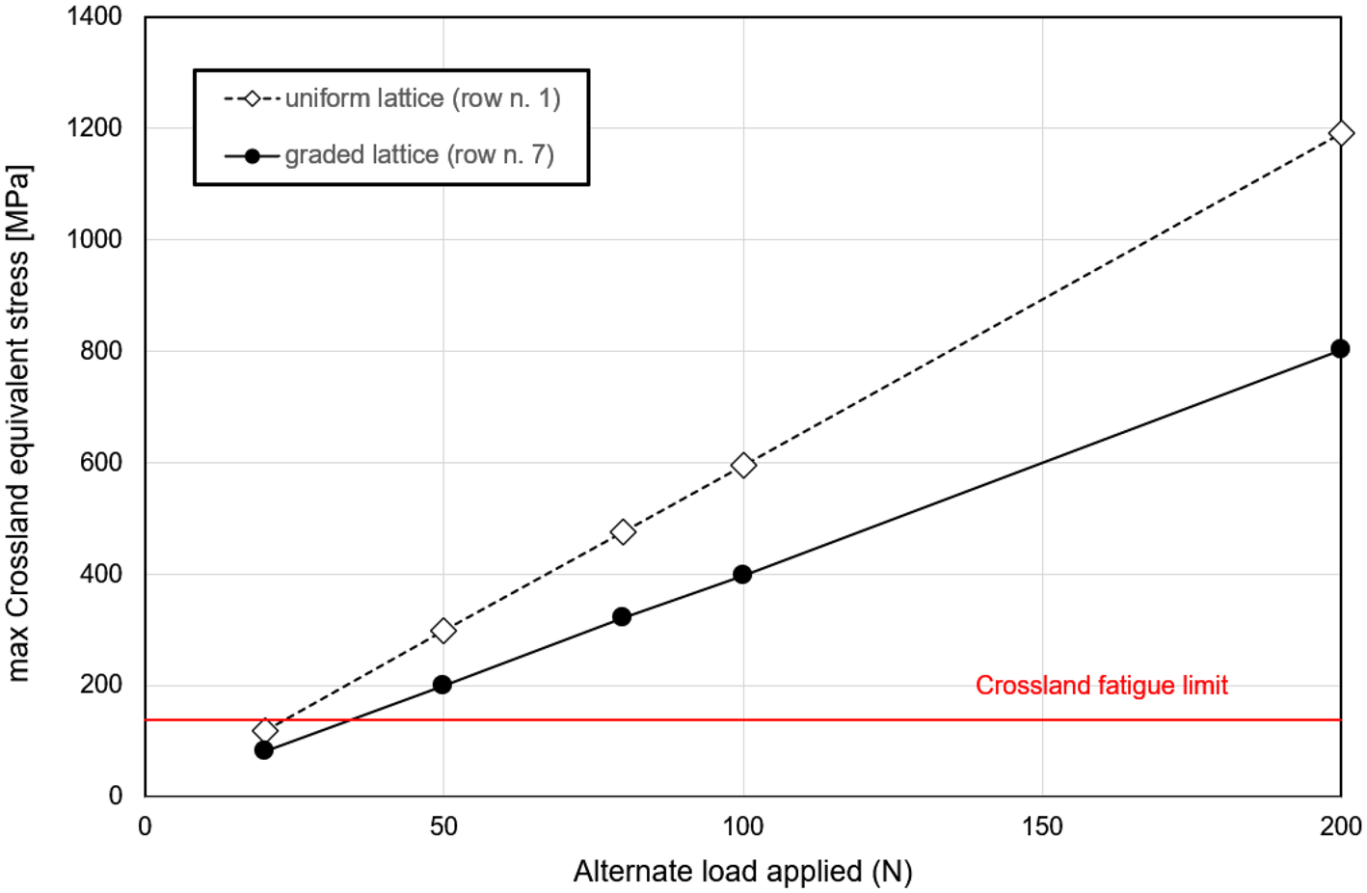
maximum Crossland equivalent stress as function of the alternate load; the two curves refers to the failure section of uniform and graded samples; horizontal line defines the Crossland fatigue limit.

The method proposed, as detailed in Sect. "Numerical results", demonstrates a high degree of reliability in predicting the occurrence of fatigue failure and accurately localizing the fatigue fracture. Consequently, it becomes feasible to compute the variation in maximum equivalent stress within the critical cell under varying loads. This, in turn, enables us to determine the maximum load that can be applied to the entire lattice structure without inducing fatigue failure. In the case of both uniform and graded samples, the maximum value of the Crossland stress within the critical cell (i.e., the cell with the highest strain tensor norm) is computed under different applied loads. Figure 11 illustrates that a force of 20 N represents the maximum allowable value to maintain local stress below the fatigue limit threshold for the samples considered.

**Figure 12:**
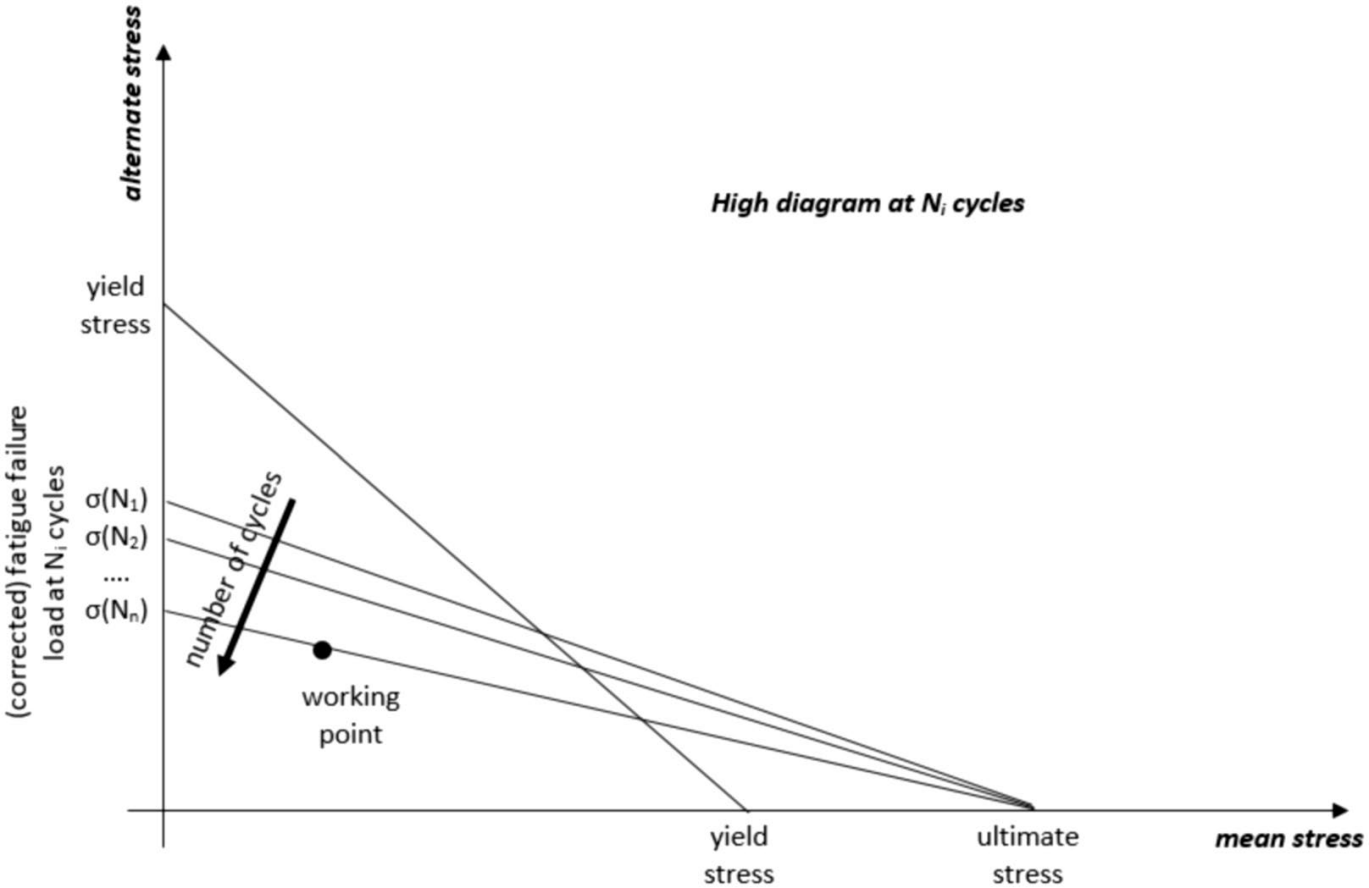
estimation of lattice fatigue lifetime through High diagrams referred to different number of cycles to failure (*N*_*i*_). In this context, distinct threshold curves are presented, each associated with varying fatigue failure loads (possibly corrected for lattice-specific characteristics). The working point identifies the threshold curve at *N*_*n*_ cycles, thereby determining the estimated fatigue lifetime. To generate the aforementioned High diagrams, it is imperative to have access to a database containing experimental values of *σ(N*_*i*_*).*

The estimation of lattice fatigue lifetime necessitates reference to a database of fatigue results derived from experimental campaigns conducted on bulk materials. These results may require corrections to accommodate the aspect ratio of lattice struts (characterized by their elongated and slender nature), the presence of notch effects, and the surface roughness typical of additive manufacturing processes. With this information available, alternative methods can be employed to estimate the equivalent stress within the critical cell, previously identified using the strain tensor norm method described in this paper. One such alternative method is the Sines criterion^56^, which facilitates the calculation of equivalent mean and alternating stresses within the critical cell. Subsequently, these two stresses can serve as coordinates for plotting a loading point within a Haigh diagram specifically constructed for a given number of cycles (N). This diagram is a function of the fatigue failure load at N cycles (potentially adjusted for the aforementioned factors), static yield, and ultimate stress. By examining the threshold curve on the Haigh diagram at N cycles, it becomes possible to assess the lattice's endurance at that specific cycle count. This process can be reiterated using additional Haigh diagrams corresponding to different cycle counts (N_i_) until convergence with a threshold curve is achieved. This convergence point provides an estimation of fatigue lifetime. A visual representation of the described procedure is presented in Fig. 12, and it should be noted that the procedure relies on a comprehensive dataset of material properties for accurate application.

## Conclusions

The proposed modeling method aims to predict the occurrence of fatigue failure in lattice structures. Unlike traditional methods, this prediction relies on a limited number of cells, specifically those with the maximum strain norm resulting from the homogenization process. By focusing computations on this small group of cells, or even a single cell in many cases, the actual three-axial stress distribution within the cell becomes significant and is converted to uni-axial equivalent stress to be compared with fatigue limit of Crossland criterion. This approach significantly reduces the computational effort required for determining the fatigue failure mode of large lattice structures. The method has undergone experimental validation using bending lattice samples featuring uniform and graded cell distributions. Remarkably, it accurately predicted fracture occurrence and location for seventeen out of eighteen samples. The strain parameter, the foundation of this model, has proven capable of accounting for the synergistic influences of external loads and cell geometry, effectively identifying the potential fracture region. Additionally, we present an approach to broaden the model's utility, enabling the estimation of the maximum applicable load without inducing failure and facilitating the prediction of service lifetime in terms of the number of cycles.

## Data Availability

Experimental results, numerical results and models are available upon request to the authors, by contacting the corresponding author at the mail address giorgio.depasquale@polito.it.

## Abbreviations

[C]: Stiffness matrix
$\left\{ {\overline{\sigma }} \right\}$: Average stress vector
$\left\{ {\overline{\varepsilon }} \right\}$: Average strain vector
$\varepsilon_{i}^{0}$: RVE strain components
$x_{i}$: Coordinate system axis indicator
$u_{i}$: Displacement in the *i* direction
$$\left[ S \right]$$: Compliance matrix
$E_{i}$: Young’s Modulus in the *i* direction
$G_{ij}$: Shear modulus in the *ij* plane
$\nu_{12}$: Poisson’s ratio *ij* plane
$$\left[ \varepsilon \right]$$: Strain tensor
$\varepsilon$: Norm of the strain tensor
$\tau_{CROSS}$: Crossland equivalent stress
$J_{2,a}$: Second invariant of the deviatoric stress tensor
$\sigma_{H,max}$: Maximum hydrostatic stress
$\tau_{f}$: Torsional fatigue limit
$$\sigma_{f}$$: Fatigue limit
$\sigma_{eq}$: Von Mises equivalent stress
$S_{i}$: Principal stress in the *i* direction
